# Does Knowledge About Physical Activity Translate into More Active Populations?

**DOI:** 10.3390/healthcare13121393

**Published:** 2025-06-11

**Authors:** Roger O’Sullivan, Aideen Sheehan, Ruth D. Neill, Toby Finch

**Affiliations:** 1 Institute of Public Health, D08 NH90 Dublin, Ireland; roger.osullivan@publichealth.ie (R.O.); aideen.sheehan@publichealth.ie (A.S.); toby.finch@publichealth.ie (T.F.); 2 The Bamford Centre, Ulster University, Coleraine BT52 1SA, UK

**Keywords:** health behaviour, knowledge, education, physical activity

## Abstract

**Background:** Physical activity has a number of benefits; however, the consequences of inactivity can have a negative impact on individuals and health and social care services. Increasing knowledge can play an important role in helping sustain behaviours that can lead to health benefits, reduce barriers and increase participation in physical activity while having a greater impact on public health policy. The aim of this study is to explore knowledge about physical activity benefits, levels of activity, awareness of risks of inactivity, sources of information about the benefits and the relationship between knowledge about physical activity recommendations and activity levels. **Methods:** A cross-sectional survey involving adults over 18 years old in Northern Ireland (weighted sample n = 2201) and Ireland (weighted sample n = 1279) was commissioned by the Institute of Public Health in 2021 to explore knowledge about health benefits and the association between knowledge and physical activity levels. Descriptive statistics in percentages were used to demonstrate the findings, while chi square tests of independence were used to examine if a significant relationship between activity and knowledge existed. **Results:** Only 4 out of 10 respondents knew the recommendations of at least 150 min weekly of physical activity. A chi square test of independence showed that the relationship between knowledge about recommendations and activity was significant (χ2(1, n = 3506) = 20.25, *p* < 0.001, not weighted). There was a sex difference in the association of knowledge about the recommended guidelines and activity levels. Women were more knowledgeable about many of the health benefits of physical activity but were less active than men. **Conclusions:** Overall, getting the adult population more active remains a challenge in public health promotion; however, knowledge alone does not equate to action. Therefore, it is essential to understand and address the range of challenges to increasing physical activity to ensure the needs of the whole population are met.

## 1. Introduction

Recommended guidelines for physical activity have been developed and adopted worldwide. These guidelines differ across population groups in terms of how much physical activity is required for good physical and mental health [1]. The international recommended guidelines (at the time of this survey) for adults, 18–64 years of age, is at least 150–300 min of moderate-intensity activity or 75–150 min of vigorous-intensity activity, with strength workouts being undertaken at least twice a week [1]. It is estimated that around three in ten people (of all ages) worldwide do not meet the recommended levels of physical activity, and there has been no improvement in global levels of physical activity since 2001 [2]. Inactivity levels are twice as high in high-income countries compared to low-income countries [2]. The rationale behind these declining activity levels could stem from a lack of knowledge around guidelines and the benefits of partaking in regular activity.

The benefits of physical activity and the challenges arising from inactivity are well established in the scientific literature and provide evidence to support that physical activity should be strongly encouraged and promoted across all age groups [1,3,4]. As acknowledged by Bull et al. [4], physical activity is a behaviour that results in benefits occurring upon increased activity and leads to improvements in cardiorespiratory fitness. This means that physical activity is a modifiable lifestyle behaviour, and therefore, changes can be made to improve this and accrue health benefits. These health benefits, alongside good health behaviours, can reduce the risk of non-communicable diseases, improve mental health, reduce hypertension, improve sleep, maintain a healthy body weight and improve cognitive function [1,3,5]. A consequence of physical inactivity is the development of serious chronic health conditions which also have an impact on healthcare utilization and systems [3,6,7]. Physical inactivity is recognized as the fourth leading risk factor for mortality worldwide, with an estimated 3.2 million deaths and 32.1 million disability-adjusted life years attributed each year to insufficient physical activity [8].

It is suggested that increasing knowledge can play an important role in helping sustain behaviours that can lead to health benefits, reduce barriers, increase participation in physical activity and have a greater impact on public health policy [9]. However, within the last two decades, research worldwide has shown that knowledge about physical activity guidelines is low [9,10,11,12]. Whilst there has been a collection of data on physical activity, there are less data available on the benefits and knowledge of recommended levels of physical activity, especially for cross-country comparisons. For example, there are no comparable data on knowledge about physical activity guidelines in Northern Ireland (NI) and the Republic of Ireland (ROI) and limited comparable physical activity statistics between the two different health systems. In NI, 25% of adults reported no activity in the previous week [13], while the Irish Sports Monitor report [14] noted that in the ROI, 36% met the guidelines. While health promotion strategies in Ireland and Northern Ireland currently target increasing physical activity (for example—Healthy Ireland [15], Active Living [16]), there is less of a focus on understanding physical activity literacy; we do not know if lack of knowledge is the reason people are not undertaking recommended levels of physical activity.

In this study, we explore knowledge about physical activity benefits, levels of activity, awareness of risks of inactivity, sources of information about the benefits and the relationship between knowledge about physical activity recommendations and activity levels in both NI and the ROI. The information in this study will provide a comprehensive understanding of whether knowledge about physical activity translates into more active populations. It is hypothesized that adults with higher levels of knowledge about physical activity recommendations will be more active than those with no knowledge about physical activity guidelines.

## 2. Materials and Methods

### 2.1. Sample and Recruitment

This was a cross-sectional study with a representative sample of adults aged in 18+ in the ROI and NI in 2021. For context, the ROI has a population of 5,149,139 [17], while NI has a population of 1,903,100 [18]. Both jurisdictions have separate governments, political systems and healthcare services. NI is part of the National Health Service that operates across the United Kingdom, whereas the ROI’s healthcare system includes both public and private healthcare services including mean- tested medical cards that provide access to a range of health services for free or at a reduced cost.

The Institute of Public Health (IPH) commissioned LucidTalk to conduct the survey within this representative sample of adults within NI and the ROI. LucidTalk Limited is a market research and polling company in NI. The project was carried out online from 19 to 28 July 2021. LucidTalk used the LucidTalk online Opinion panel (13,000+ members) for NI and the ‘Ireland Thinks’ ROI online opinion panel (30,000+). The project utilised a combined panel of over 40,000 people aged 18 and older, balanced by sex, age group, area of residence and community background, in order to be demographically representative of NI and the ROI. Sampling of the population was conducted by using a non-probability method of convenience sampling to reach all those over the age of 18 years in NI and the ROI. In NI, from the weighted sample of 2201, 56.8% were male (n = 1250) and 43.2% were female (n = 950), with the largest age group being those 45–64 years old, representing 38.8% (n = 853). In the ROI, a weighted sample of 1279 responded to the online survey, 50.4% of who were male (n = 645) and 49.6% of who were female (n = 633), with the largest age group being 45–64 years old, representing 38.7% (n = 495).

### 2.2. Survey and Procedures

The online survey contained six questions which asked about the benefits of physical activity, the length of physical activity they partook in (days per week and minutes active), sources of physical activity information, knowledge on physical activity guidelines, intensity levels required for benefits and the consequences of inactivity (Supplementary File S1). Demographic variables were collected: sex (male, female), age (18–24, 25–34, 35–44, 45–54, 55–64 and 65+), education (secondary-level education and tertiary-level education degree), socio-economic status, religion, residence area and constitutional position. The study was conducted according to the guidelines of the Declaration of Helsinki and the British Polling Council. The knowledge variable used a question on knowledge about recommendations: “How many minutes of weekly physical activity are recommended to get health benefits?” The Likert options were 30 min, 60 min, 90 min, 120 min, 150 min or more or don’t know. To identify whether there was an association between people’s knowledge about the recommendations of physical activity and their activity levels, we compared the levels of activity between those who were aware of the guideline to do at least 150 min of physical activity per week and those who were not.

### 2.3. Data Analysis

A data auditing process was carried out to ensure all completed poll surveys were genuine ‘one-person, one-vote’ responses. For NI, 2201 base samples were weighted by sex, community background and additional demographic measurements to reflect the demographic composition. For the ROI, 1279 base samples were weighted by sex, community background and additional demographic measurements to reflect the demographic composition. This methodology ensures robust and accurate balanced representative samples, reflecting the demographic composition of both NI and the ROI. The results are presented separately for each jurisdiction unless indicated otherwise, and significant differences are noted at the 95% confidence level. All data results produced are accurate to a margin of error of ±2.3% at 95% confidence. All reported margins of sampling error include the computed design effects for weighting. Descriptive statistics in percentages were used to demonstrate the findings due to the data being weighted, while chi square tests of independence were used to examine if a significant relationship between activity and knowledge existed.For the purposes of this analysis, active means physically active for five or more days per week, i.e., meeting the recommended physical activity guidelines, while inactive means not meeting the recommended physical activity guidelines, i.e., four days or less or taking part in no physical activity in a week. The data for the ROI and NI were combined for this specific analysis on knowledge and physical activity levels and not weighted. Significant differences are reported as *p* < 0.05.

## 3. Results

### 3.1. Levels and Knowledge of Physical Activity

In NI, 28% of respondents were active for 5 or more days, while this figure was 35% in the ROI, and 17% of respondents in NI and 14% in the ROI were inactive (i.e., taking part in no physical activity in a week or not meeting the physical activity guidelines). In NI, men were more likely to be active than women (31% vs. 26%); in the ROI, men were significantly more likely to be active than women (39% vs. 31%, *p* < 0.05) and less likely to be inactive (11% vs. 18%). Adults aged 65 and older were more likely to be active than adults aged 18–24 years in NI (ROI: 38% vs. 37%, NI: 33% vs. 29%). Younger adults had an overall lower level of inactivity than older adults (ROI: 39% vs. 31%, NI: 31% vs. 26%).

**Figure 1:**
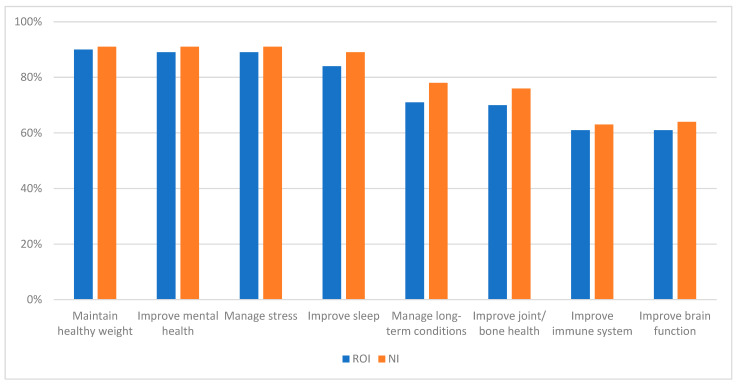
Knowledge about benefits of physical activity in NI and ROI. NI—Northern Ireland. ROI—Republic of Ireland.

Respondents had a strong awareness of the benefits of physical activity (Figure 1). Maintaining a healthy weight was the highest selected response in both (90% in ROI and 91% in NI). However, fewer people showed awareness of the benefits for the immune system (61% in ROI and 63% in NI) and for brain function (61% in ROI and 64% in NI). There was a significant difference between women and men in both locations (Table 1), with women generally more aware of the health benefits than men (*p* < 0.05).

**Figure 2:**
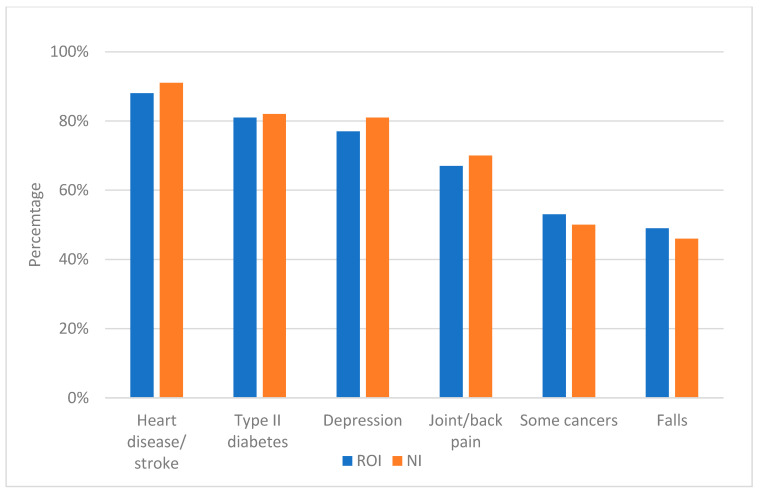
Knowledge about risks of physical inactivity in NI and ROI. NI—Northern Ireland. ROI—Republic of Ireland.

Figure 2 shows that respondents had less knowledge regarding cancer and fall risks. The majority of respondents (91% in NI and 88% in ROI) had the knowledge that inactivity increased the risk of heart disease and stroke. There was a significant difference (*p* < 0.05) in knowledge between younger and older age groups in NI and the ROI. In NI, 74% of younger people had knowledge about inactivity and cancer risks compared to 33% of older adults, while in regards to the risk of increased falls, 59% of younger adults compared to 38% of older adults had an understanding of this. In the ROI, 77% of younger people had knowledge on inactivity and cancer risks compared to 48% of older adults, while in regards to the risk of increased falls, 76% of younger adults compared to 46% of older adults had an understanding of this.

### 3.2. Knowledge About Recommended Guidelines and Intensity for Health Benefits

Knowledge about physical activity guidelines was higher in the ROI (46%) than in NI (33%). More older adults in the ROI (43%) knew the recommended guidelines compared to in NI (25%). Similarly, more younger adults (38%) in the ROI knew the recommended guidelines compared to younger adults in NI (25%). In NI, there was a significant difference between women and men in terms of knowledge about the guidelines (*p* < 0.05), with more women (37%) than men (30%) being aware of the guidelines. Under half of the highest socio-economic group in NI (37%) knew the recommended guidelines compared to 32% in the lowest socio-economic group. In the ROI, 49% of respondents in the highest education group knew the recommended guidelines compared to 41% in the lowest education group.

In NI, 64% were aware that all levels of physical activity (light, moderate, vigorous) could provide health benefits. There was a significant difference (*p* < 0.05) between men and women in terms of awareness, as women were more likely to have this knowledge (68%) compared to males (61%). There was also a significant difference between older adults and younger adults (*p* < 0.05). In the ROI, less than a third of respondents (29%) were aware that all levels of physical activity (light, moderate, vigorous) could provide health benefits. There was a significant difference (*p* < 0.05) between men and women in terms of awareness, as men were more likely to have this knowledge (34%) compared to women (25%).

### 3.3. Sources of Information

The most common source of information about health benefits of physical activity in the ROI (31%) and NI (45%) was traditional media (newspapers, TV, radio, etc.). The results highlight that social media and the workplace are important sources of information among younger adults (Table 2).

### 3.4. Association Between Knowledge About Recommendations and Activity Levels

The results show that 37% of respondents who had knowledge about the recommended physical activity levels were active five or more days per week. In comparison, 30% of those respondents who had no knowledge about the recommended guidelines were active five or more days per week. A chi square test of independence showed that the relationship between knowledge about recommendations and activity was significant χ2(1, n = 3506) = 20.25, *p* < 0.001.

### 3.5. Sex Differences

Gender breakdown from the data showed that men who were aware of the recommendations (40%) were significantly more likely to be active on 5 or more days per week than men who were not aware of them (30%). The chi square test showed a significant relationship between activity and knowledge for men (χ2(1, n = 2339) = 24.14, *p* < 0.001). However, there was no significant difference among women in activity levels between those who were aware of the recommendations of physical activity (32%) and those who did not have such knowledge (29%). A chi square test revealed no significant relationship between knowledge and activity for women (χ2(1, n = 1167) = 1.24, *p* = 0.266).

**Figure 3:**
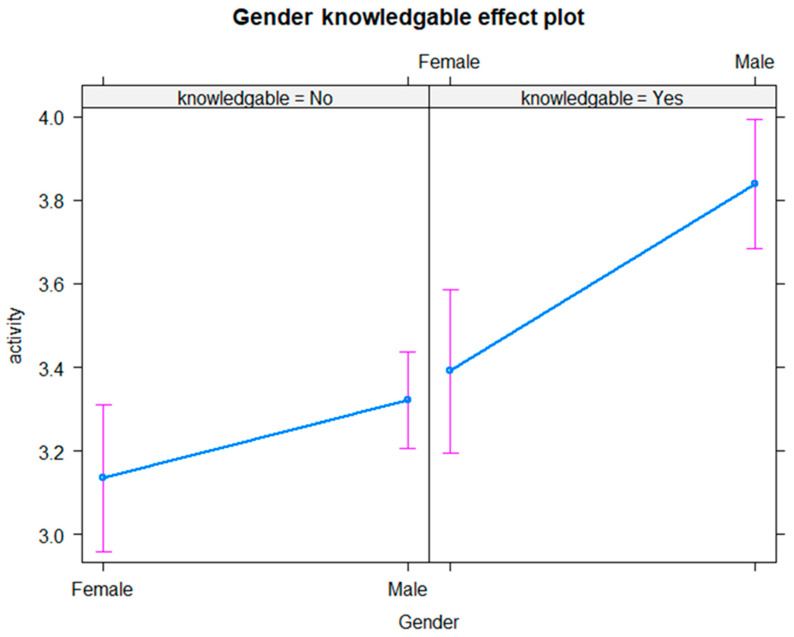
Average number of days of being active by knowledge of recommendations and gender.

A similar association was also seen when comparing the mean number of days of physical activity between those who knew the recommendations and those who did not, broken down by sex, as shown in Figure 3. The figure shows that men who had such knowledge were on average significantly more active, at 3.84 days, based on a 95% CI [3.69, 3.99], compared to men who were not knowledgeable, being active on only 3.32 days, based on a 95% CI [3.21, 3.44]. Women aware of the guidelines were active on average on 3.39 days a week, based on a 95% CI [3.19, 3.59], compared to 3.13 days, based on a 95% CI [2.96, 3.31], among women who were not aware of the recommended guidelines.

### 3.6. Educational Differences

**Figure 4:**
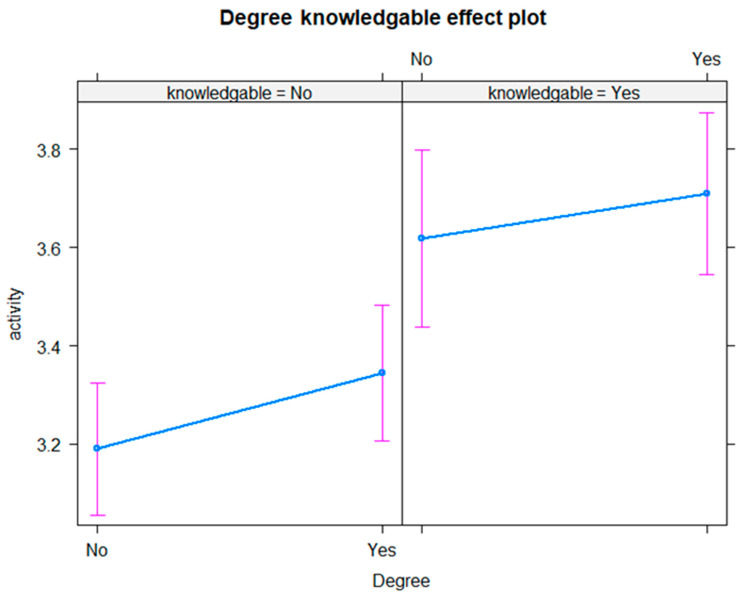
Average days of being active by knowledge of recommendations and degree status.

In a combined analysis of NI and ROI data, knowledge about the guidelines was more closely associated with higher activity than education, as shown in Figure 4. Graduates aware of the guidelines were active on 3.71 days on average, based on a 95% CI [3.54, 3.87]. There was a significant difference when compared with those who were not aware of the recommended guidelines, with graduates who were not aware of the guidelines being active on 3.34 days, based on a 95% CI [3.21, 3.48]. Non-graduates aware of the guidelines were active on average on 3.62 days based on a 95% CI [3.44, 3.80] compared with 3.19 days for non-graduates not aware of them, based on a 95% CI [3.06, 3.32], which also represents a significant difference.

A breakdown by education, **sex** and knowledge levels on an all-island basis indicates that activity levels are highest in men who have a tertiary-level degree, are aware of the guidelines and are active on 3.93 days. In women, activity levels are marginally higher in those who are aware of the guidelines, at 3.39 days, but do not vary much by education status. Overall, knowledge about guidelines is more important than educational attainment in predicting activity levels in both men and women, but **sex** is more important overall, with men generally more active than women regardless of social background.

## 4. Discussion

The present study is, to the best of our knowledge, the first comparative study to explore knowledge about physical activity benefits, awareness of risks of inactivity and knowledge about guidelines across the general population in two jurisdictions. Previous research supported the association between physical inactivity and poor health [4,19]. The aim of this study was to inform the development of public health interventions and policy, with a view to increasing physical activity at a population level, by understanding whether knowledge about physical activity guidelines and benefits differs according to demographics, where people obtain their knowledge and whether knowledge equates to higher levels of physical activity.

This survey found that despite a strong awareness of the benefits associated with regular physical activity, only around a third of the population of the ROI (35%) and NI (28%) were physically active for five or more days a week. Around a fifth of the population were inactive, i.e., taking part in no physical activity at all in a week or not meeting the physical activity guidelines. As this study was conducted during the COVID-19 pandemic, low levels of physical activity could be associated with the restrictions/lockdowns that had been implemented, which limited access to recreational facilities and altered daily routines. Comparing the evidence, the Health Survey NI [13] reported that 25% of adults were inactive, while the Irish Sport Monitor report [14] noted that 36% met the guidelines.

The results from this study suggests a significant association between knowledge and physical activity. This is a similar finding to a study in the United States (Hawaii) conducted by Heinrich, Maddock and Bauman [11], who explored the relationship between physical activity, knowledge, health outcome expectancies and behaviour. The authors found that respondents were aware that physical activity was necessary for health benefits. However, the findings in this study provide examples of knowledge about the health benefits, for example maintaining a healthy weight, improving mental health and managing stress. Overall, the combined analysis demonstrates that 37% of respondents who had knowledge about the recommended physical activity levels were active five or more days per week compared to 30% who had no knowledge about the recommended guidelines. This finding is similar to the results of a study by Bennett et al. [12], who found that recommendation knowledge was higher in those meeting the recommended guidelines versus those who did not. In the study involving a probability sample of the US population, only a third of respondents had knowledge about the guidelines (36%). An Australian study by Fredriksson et al. [8] did not find a significant association between knowledge and physical activity but found that over half of participants (55.6%) could not identify how much physical activity is recommended for health benefits. These findings indicate that further research is needed to develop a deeper understanding of population knowledge about physical activity and its relationship with physical activity behaviour and meeting the recommended World Health Organization guidelines within the context of health promotion.

The sex difference in physical activity levels between men and women has been previously well established [13,15]. In this survey, the findings show that women were less likely to be active and more likely to be inactive than men in NI and the ROI. A significant finding requiring further research is that this was despite greater awareness of the benefits of physical activity and risks of inactivity amongst women compared to men. An analysis of the association between knowledge about the recommendations of physical activity and activity levels found that there was a significant difference for men—i.e., those who were knowledgeable were more active; however, for women, this was shown not to be the case. Therefore, while women are more aware of the benefits of regular activity for health, they are less active than men, even when they have a greater awareness of the recommendations. This suggests that knowledge alone is not enough to translate into greater activity levels for women, and other barriers may exist, such as safety, time and access to suitable activities, all of which need to be examined further.

The low levels of physical activity present a significant challenge for public health across the island of Ireland. This issue is not an easy fix, and not one organization or government department can fix this alone.

Policy frameworks are in place within NI and the ROI to increase participation in physical activity (for example, Healthy Ireland [15], Active Living [16], National Activity Plan Ireland [20]). Despite these frameworks, the findings of this study indicate that we need to do more to encourage the population to be more active and to eat a better balanced diet. Less than half of respondents in the survey in the ROI and a third in NI were aware of the recommended guidelines for physical activity of at least 150 min of activity a week. This lack of knowledge is a key factor to address, as currently this is a barrier to participation in regular physical activity. However, this survey also demonstrates that knowledge about physical activity benefits is important but does not always equate to action, especially amongst women. This highlights the need to understand and address barriers (social, economic, physical, environmental) to ensure the needs and interests of the whole population are met.

### 4.1. Strengths and Limitations

This study has generated several recommendations that can inform physical activity practices and future research. However, one limitation is that cross-sectional surveys can produce a non-response bias and are only reflective of the period of time examined. Therefore, while the findings show an association between knowledge and activity, it does not prove causation, i.e., that knowledge leads to activity, and we cannot reveal causal relationships [21]. Future longitudinal studies should be carried out to address this gap. Another limitation is the different samples sizes from NI and the ROI, which can lead to much wider confidence intervals in the ROI. Online surveys are self-report in nature and, while anonymous, can be subject to social desirability, selection bias or recall bias [22]. The survey was also conducted during the time of the COVID-19 pandemic, and evidence suggests that at this time, survey fatigue was a frequent occurrence and could have had an influence on the number of respondents in this survey [23]. Finally, response options in two questions were different for respondents in NI and the ROI. These differences in options, with NI respondents having more choice, may impact the comparability of some data, particularly for questions about what level of physical activity is beneficial (light, moderate or vigorous). Respondents in NI could select more than one choice, whereas respondents in the ROI could only select one choice. The survey only asked a single question to establish whether respondents took part in daily physical activity, with the second part of the question asking about the minutes of activity. Finally, an additional limitation is that the questions used in this survey were not part of a previously validated instrument but they were extensively tested to ensure clarity reliability and validity. Future research exploring physical activity should also include an objective measure such as an accelerometer to reduce any potential measurement error from the self-reported physical activity.

### 4.2. Practical Implications

Based on the findings, three aspects can be identified as focal points of recommendations: (1) increasing physical activity especially in target groups, (2) clear and effective communication and (3) increasing knowledge about the range of benefits of physical activity and the consequences of physical inactivity. Increasing physical inactivity is a public health concern [7,24], as it can impact health (increases in mortality and non-communicable diseases and worsening mental health), the economy (increased healthcare system costs) and society as a whole (increased social isolation). Therefore, it is important to promote a culture of regular physical activity that is beneficial for people’s mental and physical health, especially as evidence suggests that physical activity levels have begun to drop-off particularly in certain groups (people with disabilities, those with chronic illnesses, older people) [14,25].

With the evidence from this study and official figures on activity levels, it is vital to provide various physical activity initiatives that suit as many people as possible. For example, providing safe, desirable, inclusive walking and cycling opportunities in local areas is crucial and has the potential to reach a wider population. The use of Health Impact Assessments in planning and policy proposals at local/national levels can help emphasise and measure potential increases in health and wellbeing associated with increased physical activity. In addition to the main report in this study [26], the IPH has published new guidance and training on Health Impact Assessments [27].

Communication is also a key element in helping increase physical activity. It is important to communicate the right message to the general public, especially to those harder-to-reach groups (people with disabilities, those with chronic illnesses, older people). Research suggests that effective communication can play an important role in changing behaviour around physical activity [28,29,30]. Simplified messages on the level of intensity of physical activity and the recommended length of time needed to achieve health benefits are essential. The findings of this study show that older people use more traditional media, while younger people use social media outlets for information. Therefore, it is imperative that messages should be communicated across both media platforms (traditional and social) to target all generations and ensure that no one is left out. Additionally, working with healthcare professionals to help spread this message can also increase communication levels as thousands of individuals use health and social care services daily. Previous research has demonstrated that healthcare professionals can play a key role in supporting and providing physical activity recommendations to service users [31]. However, healthcare professionals require training and support in this area.

Lastly, the findings of this study indicate that respondents had less knowledge regarding cancer and fall risks associated with physical inactivity, and people were also less aware of the recommended guidelines and intensity of physical activity. Based on this finding, it is clear that increasing knowledge about physical activity is important. Communicating key messages to the population can help increase their understanding of the key health benefits that can be achieved by being physically active at least five days a week for over 30 min. Benefits such as maintaining weight, managing long-term conditions like diabetes, helping reduce the risk of stroke and cancer, improving the immune system and reducing the risk of falling, particularly in older adults, all need to be promoted [1]. Increasing knowledge across the population could be achieved through engagement with schools, community groups, and traditional and new media campaigns, with videos and key messages that target all groups, even those with lower health literacy.

## 5. Conclusions

The present study is, to the best of our knowledge, the first comparative study to explore knowledge about physical activity benefits, awareness of risks of inactivity and knowledge about guidelines across the general population in two jurisdictions. This study identified that knowledge may equate to increased levels of physical activity. As hypothesised, adults with higher levels of knowledge about physical activity recommendations were more active than those with no knowledge about the physical activity guidelines. However, there are specific gender considerations and other demographic factors which can influence physical activity levels, and knowledge is only one part of this. The findings also suggest an information gap between males and females, and between older and younger adults. Therefore, future research should address why there is an information gap and the barriers to participation for women and older adults. Further research should also include qualitative studies to explore barriers to physical activity among women or older adults.

## Figures and Tables

**Table 1:** Weighted data on knowledge about benefits of physical activity in NI and ROI with key demographics.

	Total (n)	Male (n)	Female (n)	Younger Adults (18–24) (n)	Older Adults (65+) (n)	Highest Socio-Economic Group (NI)/Education (ROI) (n)	Lowest Socio-Economic Group (NI)/Education (ROI) (n)
Awareness for improving mental health, anxiety and depression							
NI	91% (2022)	88% * (1113)	96% * (908)	96% * (157)	84% * (366)	93% (894)	92% (539)
ROI	89% (1138)	86% * (554)	92% * (583)	86% (76)	81% (249)	92% (521)	83% (202)
Awareness for maintaining healthy weight							
NI	91% (2017)	90% (1132)	93% (908)	97% * (159)	82% * (356)	94% (896)	92% (539)
ROI	90% (1152)	91% (585)	90% (568)	97% * (85)	83% * (254)	95% * (536)	83% * (202)
Awareness for managing stress							
NI	91% (2005)	88% * (1107)	95% * (899)	96% * (157)	82% * (356)	95% * (907)	88% * (514)
ROI	89% (1132)	86% * (554)	91% * (579)	84% (74)	82% (251)	92% * (520)	84% * (204)
Awareness for improving sleep							
NI	89% (1976)	87% * (1089)	93% * (887)	91% (148)	85% (368)	94% * (898)	87% * (512)
ROI	84% (1075)	82% (527)	86% (548)	82% (72)	79% (241)	89% * (503)	77% * (186)
Awareness for managing conditions (e.g., diabetes/heart disease)							
NI	78% (1723)	74% * (935)	83% (788)	85% * (138)	68% * (297)	82% (782)	77% (454)
ROI	71% (913)	71% (457)	72% (456)	87% * (76)	60% (182)	75% * (423)	60% * (145)
Awareness for improving joint and bone health							
NI	76% (1675)	69% * (870)	85% * (804)	80% (131)	70% (302)	78% (746)	74% (435)
ROI	70% (897)	66% * (428)	74% * (469)	77% (67)	65% (197)	75% * (423)	60% * (145)
Awareness for improving brain function							
NI	64% (1409)	63% (794)	65% (616)	78% * (128)	50% * (219)	71% * (682)	64% * (375)
ROI	61% (779)	59% (383)	63% (397)	78% * (69)	50% * (154)	67% * (375)	53% * (128)
Awareness for improving the immune system							
NI	63% (1394)	61% (772)	65% (622)	78% * (128)	47% * (206)	71% (676)	62% * (364)
ROI	61% (784)	59% (379)	64% (405)	76% * (66)	51% * (157)	68% * (385)	50% * (120)

* Subgroup significant difference: *p* < 0.05. NI—Northern Ireland. ROI—Republic of Ireland.

**Table 2:** Sources of information about physical activity in NI and ROI with key demographics (weighted data).

	Total (n)	Male (n)	Female (n)	Younger Adults (18–24) (n)	Older Adults (65+) (n)	Highest Socio-Economic Group (NI)/Education (ROI) (n)	Lowest Socio-Economic Group (NI)/Education (ROI) (n)
Obtains information on benefits of physical activity from media (TV, radio, websites)							
NI	45% (1000)	46% (582)	44% (418)	62% * (101)	40% * (175)	47% (446)	44% (257)
ROI	31% (409)	30% (196)	34% (213)	24% (21)	44% (134)	32% (180)	35% (86)
Obtains information on benefits of physical activity from social media							
NI	40% (882)	37% (467)	44% (414)	51% (84)	28% (120)	44% (424)	42% (246)
ROI	10% (122)	8% (51)	11% (71)	19% (17)	2% (8)	10% (58)	8% (20)
Obtains information on benefits of physical activity from GP or other health professionals							
NI	26% (564)	31% * (388)	19% * (177)	39% (63)	26% (150)	21% (204)	26% (150)
ROI	11% (136)	10% (65)	11% (71)	8% (7)	14% (42)	7% (42)	12% (30)
Obtains information on benefits of physical activity from public health campaigns							
NI	38% (836)	38% (476)	38% (360)	40% (66)	41% (179)	40% (380)	37% (219)
ROI	9% (111)	7% (46)	10% (65)	8% (7)	9% (28)	10% (54)	5% (12)
Obtains information on benefits of physical activity from family							
NI	32% (714)	34% (423)	31% (290)	43% (71)	35% (153)	32% (303)	34% (200)
ROI	11% (143)	13% (82)	10% (61)	12% (11)	12% (37)	10% (54)	14% (33)
Obtains information on benefits of physical activity from friends							
NI	26% (577)	29% * (360)	23% * (217)	31% (50)	22% (95)	31% * (299)	23% * (135)
ROI	7% (86)	9% * (61)	4% * (25)	10% (9)	5% (16)	7% (20)	8% (8)

* Significant difference within subgroup: *p* < 0.05. NI—Northern Ireland. ROI—Republic of Ireland.

## Data Availability

Requests to access the datasets should be directed to Roger O’Sullivan.

## Supplementary Materials

The following supporting information can be downloaded at https://www.mdpi.com/article/10.3390/healthcare13121393/s1: Supplementary File S1: Survey questions.
