# Supplementary material for: Does Knowledge About Physical Activity Translate into More Active Populations?
Source: Healthcare (Basel). 2025 Jun 11;13(12):1393. doi: 10.3390/healthcare13121393 (PMC12193354; doi:10.3390/healthcare13121393)
Supplement: Supplementary file 1 [file healthcare-13-01393-s001.zip › healthcare-3632283-supplementary.pdf]

Supplementary File S1: Survey Questions

1. Physical activity. “During the last week (7 days), on how many days did you take part in physical activity that raised your heart rate and caused you to breathe somewhat harder than normal?

1a). Physical activity: "You've indicated you did some activity during the last week On the days that you were active, how long were you active for on average?"

2. Physical activity, Your Knowledge: "How many minutes of weekly physical activity are recommended to get health benefits?"

3. Benefits of physical activity: "Being physically active helps to…" Please select all that apply

- Improve mental health, anxiety and depression
- Maintain healthy weight
- Manage stress
- Improve sleep
- Manage long term health conditions such as diabetes and heart disease
- Improve joint and bone health
- Improve brain function
- Improve your immune system

4. Intensity. “What intensity of physical activity benefits health?”

- Light intensity (e. g. housework, yoga)
- Moderate intensity (e.g.walking, cycling)
- Vigorous intensity (e.g. dancing)
- All of the above
- Don’t know/ not sure

5. Consequences of inactivity: “Being physically inactive increases your chances of…”

- Heart disease and stroke
- Type II diabetes
- Depression
- Joint and back pain
- Some cancers
- Falls
- Don’t know/ not sure

6. Sources of information: “Think about the last time you heard about the benefits of being physically active, what was the source of this information?”

- Media – TV, Radio, Newspapers, Online
- Social Media – Twitter, Facebook, Influencers
- Public Health Campaign
- Family
- Friends
- GP or other health professional (e.g. Nurse, OT, Physio)
- Workplace
- Books, Journals or Publications

- Coach/Gym Instructor
- Fitness Centre/Community Centre
- School
- University
- Other
- Don’t know/ Not sure
